# Tailoring Self-Help Mindfulness and Relaxation Techniques for Stroke Survivors: Examining Preferences, Feasibility and Acceptability

**DOI:** 10.3389/fpsyg.2019.00391

**Published:** 2019-02-26

**Authors:** Xu Wang, Connie Smith, Laura Ashley, Michael E. Hyland

**Affiliations:** ^1^Leeds School of Social Sciences, Leeds Beckett University, Leeds, United Kingdom; ^2^Birmingham and Solihull Mental Health NHS Foundation Trust, Birmingham, United Kingdom; ^3^School of Psychology, Plymouth University, Plymouth, United Kingdom

**Keywords:** stroke, anxiety, distress, feasibility and acceptability, self-help intervention, mindfulness and relaxation technique, tailored technique

## Abstract

**Objective:** Studies on psychological techniques to reduce stroke-related anxiety and/or distress are limited. More scarce is research on tailoring such techniques to suit stroke survivors’ needs, including the needs of those with aphasia. To address this gap, we report two sequential studies. Study 1 explored preferred psychological techniques (i.e., mindfulness and relaxation) and ways to modify them for stroke survivors, including those with communication difficulties. Study 2 examined the feasibility and acceptability of these modified techniques with a new sample of survivors.

**Design:** Mixed-methods using qualitative and quantitative approach in both studies.

**Participants:** All participants were stroke survivors living in the community (Study 1: *n* = 13, median age = 61 years; Study 2: *n* = 38, median age = 67 years).

**Interventions and Procedures: Study 1:** seven techniques representing commonly used types of mindfulness and relaxation were filmed on a professionally produced DVD. Participants feedback on how these techniques could be tailored to meet their needs, their preferences for techniques and reasons for likes and dislikes.

**Study 2:** four favored techniques from study 1 were modified and re-filmed into a new DVD. A new group of participants were asked to practice them twice daily, five times a week for at least 4 weeks. They completed questionnaires at the start of the study (T1), returned approximately 4 weeks later completing the same measures (*n* = 24 at T2). Focus group discussions/interviews were conducted at the end of T2 exploring the feasibility and acceptability of these techniques.

**Results:** Four techniques were favored by participants in Study 1. After adaptation, these techniques were generally perceived as acceptable, user-friendly and beneficial to participants who participated in the focus groups /interviews in Study 2. A ‘once a day’ practice frequency could make practicing more feasible. Participants also preferred having choices- multiple techniques could be more useful than single technique.

**Conclusion:** Tailoring psychological techniques for stroke survivors is beneficial. Tailored techniques in a self-help DVD format seemed feasible and acceptable, however, a less frequent practice would be easier for stroke survivors. Future studies should seek to recruit a more heterogenous sample as well as implementing strategies to increase the retention rate.

## Introduction

Anxiety is common following stroke and seems to persist and worsen over time. Approximately 24% of stroke survivors are affected by clinically diagnosed anxiety 6 months or more after the stroke (Campbell Burton et al., 2013). Ten years following a stroke, the prevalence rate ranges from 32 to 38% (Ayerbe et al., 2014). Stroke survivors may also experience distress which may not meet a clinical diagnosis of anxiety (Townend et al., 2006b; Gilworth et al., 2009; Campbell Burton et al., 2013). This anxiety and distress negatively impacts on stroke survivors’ daily living, quality of life and confidence in social participation (Hare et al., 2006; Campbell Burton et al., 2013; Ayerbe et al., 2014; Horne et al., 2014). At present, studies on psychological interventions to reduce stroke-related anxiety and/or distress are limited (Intercollegiate Stroke Working Party [ISWP], 2016; Knapp et al., 2017). Even more scarce is research on tailoring such interventions to suit stroke survivors’ needs, including the needs of those with aphasia. This patient-centered approach is recommended by the latest United Kingdom National Clinical Guideline for Stroke (Intercollegiate Stroke Working Party [ISWP], 2016; Andrew, 2017).

Among the few studied psychological interventions in people after a stroke, mindfulness and relaxation techniques have been shown as useful self-administered methods to alleviate anxiety and tension after stroke (Lazaridou et al., 2013; Golding et al., 2015). Mindfulness is thought to reduce distress through decreasing rumination and improving attentional control. It shifts and re-directs attention to the current moment rather than thinking about past or future worries (Jain et al., 2007). Relaxation is considered to work by generating a psychophysiological state of decreased arousal that counteracts the stress response (Benson and Klipper, 2000). The effects of mindfulness and relaxation can be achieved and enhanced through regular practice (Langer, 1989; Benson and Klipper, 2000).

A systematic review of mindfulness-based intervention (MBIs) following stroke suggested that MBIs may generate a range of benefits including reduced anxiety in this population (Lawrence et al., 2013). A recent acceptability study reported that impairment from stroke and length of the session (2 h) could be barriers to participate in mindfulness sessions for stroke survivors and caregivers (Jani et al., 2018). The mindfulness sessions in the above literature, however, were not administered as self-help interventions; they were delivered by mindfulness teachers or practitioners. Yoga is a form of mindfulness which can be used as self-help practice in stroke rehabilitation. A recent Cochrane systematic review suggested that yoga could help reduce state anxiety and had a positive effect on at least one aspect of stroke survivors’ quality of life (Lawrence et al., 2017).

Self-help relaxation could be a practical and inexpensive intervention for post-stroke anxiety. Golding et al. (2015) provided evidence supporting this concept. Stroke survivors who participated in their pilot study completed the Hospital Anxiety and Depression Scale- Anxiety Subscale (HADS-A) during the initial screening and again, each month across 3 months. Survivors in the intervention group received the autogenic relaxation via a self-help CD and were asked to practice the relaxation five times a week for a month. A significant reduction in anxiety at each time point (Months 1, 2, and 3) was found among participants in the intervention group as compared to those in the control group (without relaxation practice).

Although there are various types of mindfulness (e.g., yoga, meditation) and relaxation (e.g., progressive muscle relaxation, autogenic relaxation), past studies tend to focus on a single type at a time (Chan et al., 2012; Streeter et al., 2012; Golding et al., 2015). It is recommended that psychological stress-management interventions should include multiple techniques as opposed to a single component (Spence et al., 1999). Additionally, self-help materials should be user friendly and suit users’ needs (Richardson et al., 2008). Past studies, however, did not test whether self-help mindfulness or relaxation is suitable or should be tailored to stroke survivors’ needs. In fact, existing mindfulness and relaxation techniques are often poorly optimized for stroke survivors, especially those with communication difficulties (Lazaridou et al., 2013; Intercollegiate Stroke Working Party [ISWP], 2016). People are more likely to adhere to and practice techniques that are tailored to their needs and preferences (Hyland et al., 2016).

We report two sequential studies in this paper. Study 1 aimed to explore stroke survivors’ preferred mindfulness and relaxation techniques delivered on a self-help DVD, and ways in which these techniques could be modified to better suit their needs. The techniques favored by participants in study 1 were modified and a new ‘bespoke’ DVD was subsequently made. Study 2 was conducted to examine the feasibility and acceptability of these modified techniques delivered on a DVD.

## Study 1 Exploring Stroke Survivors’ Preferred Mindfulness and Relaxation Techniques

### Methods

#### Participants

Using convenience sampling methods, a total of 13 participants were recruited via Stroke Association community support groups and a Speakability group in the North and West Yorkshire, United Kingdom. Speakability groups are support groups ‘run by and for people with Aphasia’ following stroke or other neurological conditions (“The Stroke Association,” n.d.). The inclusion criteria for participation were: (1) formally diagnosed with a stroke when initially admitted to hospital; (2) have since been discharged from hospital to live in the community; and (3) aged above 16+. Our study did not exclude patients with communication difficulties unlike many other studies (Townend et al., 2006a). No specific additional exclusion criteria were imposed. Twenty-five participants were contacted initially and 12 refused to participate. Five stated that they were not interested in research whilst the rest did not provide their reasons.

#### Material- the Techniques, the Discussion Guide and Measures

Seven techniques were selected to represent the variety of commonly used forms of mindfulness and relaxation: Breath watch; Body relaxation; Counting; Word repetition; Positive emotions; Thinking of a nice place; and Body movement. These techniques had been used among people with other medical conditions (Hyland et al., 2016). In line with this previous study, techniques were filmed on a 20-min professionally produced DVD. A demonstrator instructed how to perform each technique followed by a 1-min practice time in-between. The description of these techniques is listed in Table 1.

**Table 1:** Description of the original 7 techniques used in Study 1.

Name of the technique	Description
**Breath watch** (mindfulness)	Focuses on breathing and noticing their breath as they breathe in and out. They were asked not to change their breathing, rather just to watch it happen.
**Body relaxation** (autogenic relaxation)	Focuses on different parts of the body and concentrating on relaxing that part. Participants do not need to physically move any body parts
**Counting** (mindfulness- mantra meditation)	Where participants mentally count numbers (e.g., 1, 2, 3,4, 1, 2, 3, 4) in their head
**Word repetition Positive emotions** (relaxation- guided imagery)	Is similar to counting but repeating a meaningless word in one’s head
**Thinking of a nice place** (relaxation- guided imagery)	Ask participants to generate a positive emotion experience by imaging a ball of light filling them with rays of happiness and love Ask participants to imagine a place where they were happy in the past. This technique and positive emotion also incorporated principles from positive psychology which involve mental exercises that cultivate positive mood states (Seligman et al., 2005)
**Body movement** (Mindfulness- Kundalini Yoga)	Focus on small bodily movements (e.g., raising or lowering one’s hand or even just a finger)

A focus group discussion guide was used to explore the preference between survivors in applying these different techniques and to obtain views on how these techniques could be tailored to meet survivors’ needs, including the needs of those with communication difficulties. A sample question and follow-up prompts were: How do you feel about using these techniques? How easy did you find it (prompt)? Tell me a bit on why you found it easy/difficult (prompt)?

Two short questionnaires were used to measure participants’ preference of the techniques. One was to rate each technique using a score of 1 (least positive) to 10 (most positive). The other asked participants to rank the seven techniques in order of preference, with 1 representing the technique they liked the best.

#### Procedure

Ethics approval was granted by the first author’s institutional research ethics committee. All participants gave written consent before participating. Four focus groups were conducted lasting approximately 1.5 h each, which included two, two, four and five participants, respectively. All group discussions were run by the first author (XW) and held in a meeting room with a computer and projector in her institution. There were several breaks during each meeting in order not to exhaust participants. Participants completed a short demographic questionnaire and their diagnosis of stroke. They then watched the DVD (via a projector) and practiced each technique following the instructions. To reduce order effects for the groups, the order of presentation of the techniques were presented in three different orders, two of which were reversed on the DVD. Following practicing, participants discussed their opinion of using these techniques, their preferences and reasons for their likes and dislikes. All discussions were digitally recorded and the discussion guide was used in all. Participants also completed the questionnaires at the end of the meeting. Each participant received a £15 shopping voucher as a token of appreciation for their time.

### Results

#### Sample Characteristics

Participants were 13 stroke survivors (8 males) with a median age of 61 years (range: 51–76). Twelve of the 13 survivors had only one stroke. At least five participants (38%) had communication problems as they were recruited from a Speakability group which is run by and for people with aphasia only (“The Stroke Association,” n.d.). The time between their stroke and the commencement of the project ranged between 5 and 16 years, with a median length of 10 years.

#### Preferred Techniques and How to Tailor Them Further to Suit Stroke Survivors’ Needs

The median and interquartile range of participants’ ratings are reported in Table 2. Thinking of a nice place and breath watch received highest rating scores, followed by positive emotions and body relaxation. The ranking score showed similar patterns, with ‘nice place’ as the most favorite, followed by breath watch, positive emotion and body relaxation.

**Table 2:** Median and interquartile range of rating and ranking scores of 7 relaxation techniques (Study 1: *n* = 13).

	Rating scores (range 1–10)		Ranking scores (range 1–7)	
	
	Median	IQR	Median	IQR
Counting	2	1–4.5	7	4.5–7
Word repetition	2	1–7	6	4.25–6
Body movement	5	3–7	4.5	2.25–5
Body relaxation	7	7–9.5	4	2.25–5
Breath watching	9	5.5–10	2	1–2.5
Positive emotions	8	1–9.5	3	2.5–5
Thinking of a nice place	9	8–10	1	1–2.5

*A rating score of 10 was the highest whereas a ranking score of 1 indicated a top rank.*

The recorded focus group discussions were transcribed verbatim by AC and checked against the recordings for accuracy to eliminate errors by XW. Thematic analysis was carried out using the steps defined by Braun and Clarke (2006). Several themes were developed based on the data to describe participants’ preferences of and suggestions of ways in which these techniques could be tailored to suit their needs. Selected quotes and overall descriptive themes exploring participants’ preference of and recommendations are listed in Table 3.

**Table 3:** Study 1_Selected quotes and overall descriptive themes representing participants’ preference and suggested modifications.

Themes	Subthemes	Sample quotes
Feelings generated from practicing the techniques		*I was ehh, it* (***thinking of a nice place***) *took me back to thinking about a wedding in Bali on an elephant. Then it brought a tear to my eye, it was a good one, yea.* (P2, Male, 60) *I know I am relaxed when I can open and close my left hand… because of the work we are doing* (***body relaxation***), *so I am fairly relaxed at the moment because I can open and close my hand.* (P3, Male, 69) *It is because normally we don’t stop, we are always doing something else, but I think this* (***breath watch***) *is fantastic...* (P4, Male, 63, with communication problems) (***Word Repetition***) *Didn’t do anything. Well you could just erm, you could just repeat the word automatically, without it really uh doing anything, well that’s what I found…nothing happened really.* (P10, Male, 56)
Perceived ease or difficulty of using the techniques	Most techniques were simple and easy to follow and practice	*I think it* (***breath watch***) *goes across the border that anyone can do this whether they are disabled or not.* (P3) *This* (***positive emotions***) *is very very, very easy instructions to follow.* (P5, Female, 60, with communication difficulties)
	Some techniques were difficult to use or did not work	*That* (***word repetition***) *was like a mantra, wasn’t it? I was um having difficulty for that. Um.. I don’t know what word (to say).* (P7, Female, 51, with communication difficulties) *I think* (***counting***) *it’s difficult because I have to concentrate. Um…that is what um a lot of stroke people find that the concentration goes…* (P6, Female, 61, with communication difficulties) *That uh exercise* (***body movement***), *that uh, I’ve a, every day in the bathroom uh cleaning the kitchen and all, all sorts vacuuming all sorts that’s no good that.* (P12, Male, 56)
	Liked the DVD format of delivering the techniques	*I think that DVD overall will be better, it’s easier to follow somebody describing the step than trying to decipher what the meaning behind the words is. You can pause it (the DVD) and you can go back you know, you, repetition it is more versatile than the written page.* (P3)*Show it on a computer like you, I would look on it — visually, but I cannot write.* (P4)
Modifying the techniques to suit stroke survivors’ needs	Change some wording	*To emphasize ‘think about’ it (body relaxation) you know. Just concentrate on that side and move to the other side without moving.* (P11, Male, 65)
	Required modifications for survivors with aphasia	*I heard the color ‘yellow’, and just concentrate on that…I get confused about colors, still…maybe say ‘warm and fuzzy feeling’.* (***Positive emotion****).* (P7) *I would like him to talk when it was quiet (during the practice time), tell me, prompt me… also put it (wording) on screen in writing.* ***(Body relaxation***) *…Just taking into consideration the aphasic point about pictures, and for P8 (who has severe communication difficulties), to speak as slowly as possible in the video*. (P5)
	Recommendations to make the DVD look better	*I would say DVD but delivered by someone who is sitting down which is a visual way of relaxation. Someone that is sat down somewhere that looks relaxing anyway, as opposed to someone who could be stood at the blackboard at school*. (P2)

#### Selecting and Modifying Techniques

Findings suggested that stroke survivors preferred four techniques among a set of existing techniques. They perceived the four techniques as ‘easy-to-do’ and commented on their effect of relaxing. “Body movement,” “counting” and “word repetition” were least preferred and considered not suitable for stroke survivors especially those with communication difficulties. They were subsequently removed in the modified technique DVD used in Study 2.

**FIGURE 1:**
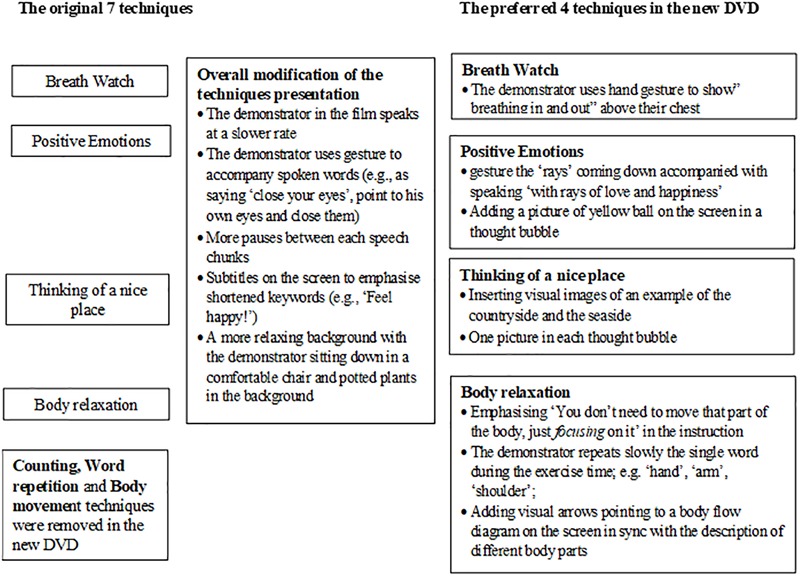
How the new DVD presents the 4 preferred techniques with required modifications.

**FIGURE 2:**
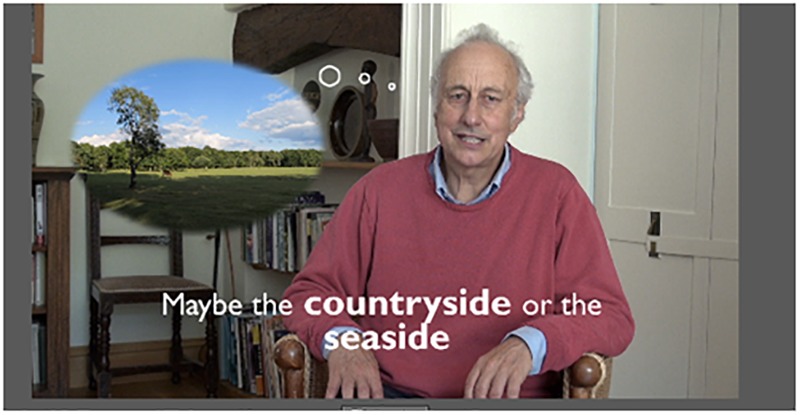
A snapshot of one of the re-filmed scenes on the DVD.

We modified the four most preferred techniques and produced a new DVD based on participants’ feedback. For example, in ‘body relaxation,’ we highlighted that participants just need to focus mentally on different parts of the body and concentrate on relaxing that part of the body. This emphasis enables survivors with apraxia to perform ‘body relaxation’ as it does not involve actual motor movement or physical practice. To make the techniques more suitable for people with aphasia, we worked with a qualified speech and language therapist, who is also specialized in working with people post-stroke, using the supported conversation’ principle to facilitate comprehension of the techniques and its instructions (Kagan, 1998; Pound et al., 2008; Jayes and Palmer, 2014). Figure 1 describes the details of these modifications. Figure 2 is a snapshot of one of the scenes of the revised ‘thinking of a nice place’ technique. This new DVD is used in Study 2.

## Study 2: Exploring the Feasibility and Acceptability of the Modified Techniques

After modifying the four preferred techniques, we aimed to assess whether stroke survivors would perceive them feasible and acceptable on a DVD format. Feasibility included perceived acceptability and practicality of performing these techniques. Study 2 had two phases: Time 1 (T1) and Time 2 (T2).

### Methods

#### Research Participants

Stroke survivors were recruited through 24 community stroke support groups in West Yorkshire and the West Midlands. CS attended the group meetings to introduce the study or sent the group leader a brief introduction to allow their members to contact her if they were interested in participating. The inclusion criteria required participants to: (1) have received a formal diagnosis of stroke; (2) have been discharged from hospital to live within the local community; (3) be aged 18 or over and; (4) be English literate. Similar to Study 1, we did not exclude survivors with communication difficulties unlike many other studies (Townend et al., 2006a). However, people were excluded if they felt that they had any medical, physical or cognitive conditions that may affect their capacity to consent or participate in this study. The sampling was opportunistic, with survivors being approached and asked to volunteer during their group session or after the session.

#### Measures and Apparatus

The four mindfulness and relaxation techniques favored by participants in Study 1 were modified in line with their suggestions: (1) positive emotion; (2) body relaxation; (3) thinking of a nice place; and (4) breath watch. They were re-filmed into a 15-min-long new DVD which begins with a brief introduction on the benefits of psychological techniques to promote survivors’ engagement (Kneebone et al., 2014). We also provided participants with a brief instruction on using the techniques at home, which suggests how they could prepare for the relaxation and mindfulness (i.e., allow themselves 15–20 min in a quiet place free from disturbances), what they need to do in terms of inserting the DVD and following the on-screen instructions, and finally how they end the relaxation session (i.e., outlining at the end they would hear a beep).

Participants’ post-stroke functional ability and independence, situation-specific confidence and level of anxiety were measured by the 10-item Barthel Activities of Daily Living Index (Collin et al., 1988), the Daily Living Self-Efficacy Scale (DLSES) (Maujean et al., 2014), and the Hospital Anxiety and Depression Scale anxiety subscale (HADS-A) (Zigmond and Snaith, 1983), respectively. These rating scales have been previously validated for use among stroke survivors (Aben et al., 2002; Maujean et al., 2014).

The Barthel ADL Index yields a total functional ability score. Total scores range between 0 demonstrating a less severe disability, and 20 indicating a more severe disability. Wade and Hewer (1987) also categorized people’s functional ability into five categories according to the total score: very severely disabled (0–4); severely disabled (5–9); moderately disabled (10–14); mildly disabled (15–19) and independent (20). Participants completed the Barthel Index at T1 only.

The 12-item DLSES consists of two subscales: psychosocial functioning and activities of daily living. Participants responded using a 0–100 scale with 10-unit intervals, whereby they rated their degree of confidence at performing each task. For example, ‘0’ = ‘cannot do at all,’ ‘50’ = ‘moderately can do,’ and ‘100’ = ‘highly certain can do.’ An overall DLSES score can be obtained by combining the score of each item, dividing this total by the number of items (12). A higher score indicated higher self-efficacy/confidence (Maujean et al., 2014).

The HADS-A consists of 7 items scored on a 4-point Likert scale (ranging from 0 to 3). The total score of HADS-A ranges from 0 to 21, with higher scores indicating higher levels of anxiety. A cut-off value of 8+ for HADS-A indicates possible cases for anxiety disorders (Bjelland et al., 2002) on overall anxiety scores.

Socio-demographic and Clinical Data such as participants’ age, gender, total number of strokes and time since their last stroke was also collected. The Brief Illness Perception Questionnaire (B-IPQ) (Broadbent et al., 2006) was also completed, but it is not analyzed and reported here.

A self-report practice diary was used to measure participants’ engagement of the techniques at home. The tick-box diary had three options- ‘did not practice today,’ ‘practice once today’ and “practice twice today.”

A discussion guide/ interview schedule was used to explore participants’ perceptions and experiences regarding the feasibility and acceptability of practicing these techniques.

#### Procedures

Ethics approval was granted by the first author’s institutional research ethics committee. Local group leaders’ consent was obtained prior to approaching their members. All participants gave written consent before participating. The data collection was conducted at where the support group met at two time points - T1 and T2. T2 was approximately 4–6 weeks after T1, depending on when the support group’s next meeting was scheduled.

Participants completed the questionnaires individually within their group setting, followed by practicing the techniques together using the DVD. Participants watched the DVD and practiced all four techniques following the instructions. At the end of T1 they were given a copy of the DVD, the home practice instructions, and the practice diary to take home. They were asked to practice the techniques twice a day, 5 days a week between T1 and T2. This was in line with suggestions by Benson and Klipper (2000), that frequent relaxation practice (i.e., twice daily, five times per week) maximizes the potential outcomes. About 2 weeks after T1, CS contacted participants to answer any queries regarding their practice and to remind them of the date of T2. Each participant received approximately 10 min of telephone contact. None of the participants sought additional telephone contact or help during the study.

Participants completed HADS-A and DLSES, watched and practiced all four techniques together in their support group setting for a second time at T2. It was due to practical reasons and convenience for participants that they practiced together in their group, as opposed to practice individually. After a short break focus group discussions or semi-structured interviews were conducted (if a participant preferred being interviewed). Three focus groups (an average of 50-min long) and two semi-structured interviews (20-min long) were conducted. CS ran all of the group sessions and interviews using the discussion guide/interview schedule. All discussions and interviews were audio-recorded with participants’ consent.

#### Data Analysis

Feasibility was considered in terms of retention and adherence rate (Eldridge et al., 2016); using the practice diary data, which were analyzed mainly descriptively. The median number of practices was calculated based on a 4-week timeframe as this time gap was most common between T1 and T2. We also calculated median and interquartile range (IQR) for measures used at T1 and T2.

The audio-recording of qualitative data were transcribed verbatim by CS and a selection of transcripts were checked for accuracy to eliminate errors by XW. Thematic analysis was carried out using the steps defined by Braun and Clarke (2006) to report key themes relating to acceptability and the practicality of delivery element of feasibility. The datasets were coded by giving every meaningful piece of text a descriptive label indicating the meaning of that piece of text. The next step involved grouping those pieces of text which had been coded similarly, and applying a theme label to those text to capture a pattern. At the end of this process we arrived at four themes concerning participants’ views on the acceptability and feasibility of these techniques delivered on a DVD.

### Results

#### Recruitment, Sample Characteristics and Retention

Thirty-eight stroke survivors completed the quantitative measures at T1. At least nine participants (24%) had communication problems as they were recruited from Speakability groups which are support groups for people with aphasia only. Eighteen people who also volunteered to participate were excluded due to either: (1) no formal diagnosis of stroke (*n* = 17), or, (2) having post-stroke memory impairment therefore could not consent nor participate (*n* = 1).

**FIGURE 3:**
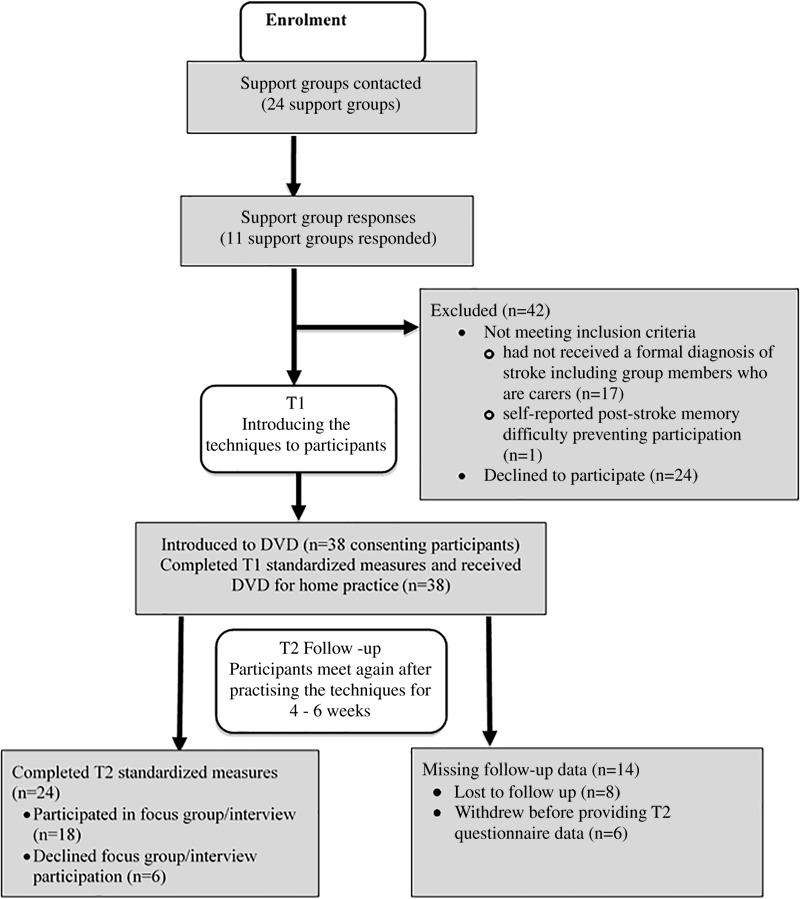
Flow diagram showing procedure of recruitment, participation and follow-up.

Twenty-four stroke survivors completed the quantitative measures at T2. The retention rate for T2 was 63.2%. Eighteen (two with communication difficulties) of the 24 participants took part in the focus group discussions or semi-structured interviews. Figure 3 shows a flow diagram for Study 2.

All participants reported themselves as White British. Nearly one fifth of participants had their stroke within the last year, and 60% had their strokes over 4 years. Participants’ median functional ability was ‘mildly disabled’ with their Barthel ADL scores ranging from 6- ‘severely disabled,’ to 20- ‘independent.’ Survivors had a moderate to a high level of overall self-efficacy, activities of daily living and psychosocial functioning at both T1 and T2. Participants’ median anxiety scores were almost ‘borderline abnormal’ at both T1 and T2. Table 4 presents participants’ demographic data, stroke characteristics, DLSES and HADS-A scores at T1 and T2.

**Table 4:** Demographics, stroke characteristics; HADS-A and the Daily Living Self-Efficacy Scale (DLSES) scores at T1 and T2.

	T1 (*n* = 38)	T2 (*n* = 24)
	Median and IQR	Median and IQR
Barthel total score,	16 (14–19)	–
Age, (years)	67 (57–72.5)	64.5 (55.8–70.8)
Time since stroke (years)	5.5 (1.5–10)	4.3 (1.4–9.8)
HADS-A total score	7 (5–10.5)	8 (6.3–11)
The DLSES total score	65 (41.9–77.1)	72.5 (54.8–79)
Psychosocial functioning subscale	69.1 (45.9–78.4)	73.8 (54.1–87.5)
Activities of daily living subscale	57.5 (33.1–80.3)	67.5 (52.5–82.5)

	**n (%)**	**n (%)**

Gender- male,	18 (47.4)	11 (45.8)
Stroke history		
First-ever stroke	29 (76.3)	17 (70.8)
Second stroke	8 (21.1)	6 (25)
Five strokes	1 (2.6)	1 (4.2)
Anxiety disorder caseness (HADS-A ≥ 8)	18 (47%)	14 (58%)
Physical disability (Barthel score)		
No physical disability (20)	4 (10.5)	3 (12.5)
Mild impairments (15–19)	20 (52.6)	13 (54.2)
Moderately disabled (10–14)	12 (31.6)	7 (29.2)
Severe disability (5–9)	2 (5.3)	1 (4.1)

We conducted an attrition analysis and found no significant differences between non-participants (*n* = 14) and participants at T2 quantitative measures (*n* = 24) in terms of age, time since stroke, functional ability and self-efficacy. However, those who did not participate at T2 were significantly less anxious at T1 [*t*(36) = -3.73, *p* = 0.001).

#### The Frequency of Practice – Diary Data

Fourteen of the 24 participants at T2 (58%) returned their practice diaries, among which one was blank. Unfortunately, we did not know why this participant had his/her diary blank, nor did we know the reasons why others did not return their diaries, despite participating at T2. A further one participant recorded 1 week’s practice only as she preferred listening to music as a way to relax. Thus, we had 12 participants’ diary data included in the analysis. The median practice frequency was 27 times over 4 weeks (range = 5–45; interquartile range = 11–39), which indicated that participants practiced about once every day over 4 weeks. The total number of practice times were not correlated to participants’ characteristics such as age, time since stroke, Barthel scores, HADS-A or confidence scores at T1 and T2.

#### Qualitative Focus Group/Interview Data

Four themes were generated concerning the acceptability and feasibility of these mindfulness and relaxation techniques delivered on a DVD. They were: (1) the techniques themselves are user-friendly; (2) difficult to practice so often; (3) techniques are beneficial and useful; and (4) intention to keep using the techniques in future. These themes and selected quotes are presented in Table 5.

**Table 5:** Acceptability and feasibility of the modified techniques: selected quotes and overall descriptive themes from Study 2.

Themes	Sample quotes
The techniques themselves are user-friendly	*I can slot straight into it [using the techniques] now, even without the person on the DVD… I find the more you do it, the easier it gets to do.* (CA6, Female, 63) *Yes, think they are easy and simple. You don’t have to move I can just sit there…I can just sit like I would watching TV, turn the DVD on and I don’t need to concentrate on doing anything whilst I am sitting.* (SH4, Male, 75) *The way it [the tailored techniques] was put over was very good… cause it gave you a selection for you to choose from. If you didn’t like one you could try another sort of thing.* (CA1, Male, 70) *I don’t make the time… I’m always on the go so I never get time to.* (ST2, Female, 45)
Difficult to practice so often	*It doesn’t sound much when you say 15 min, twice a day, but actually sitting down to do it and getting yourself ready makes it take longer… And it was only when I had done it so many times, with the difficulty of fitting it in around all the other stuff, that I felt confident enough [to practice].* (BR4, Female, 67, with communication difficulties) *He (BR5, Male, 53, with communication difficulties) was fairly overwhelmed with the instruction to try and practice the techniques twice a day, five times a week... If it had been the case of practicing once a day, he may have been more motivated to practice.* (BR5′s carer, conversation during telephone follow-up)
Practicing these techniques are beneficial and useful	*It’s been very beneficial and I feel a lot calmer as a result. It has also improved my confidence … It helps take your mind away and just be calm.* (CA6) *Since I’ve been making the time to relax it does calm me down when I get worked up about the stroke and things.* (ST3, Female, 46) *Well, when I feel stressed that is when I don’t feel very confident. So like what I was telling you about my car and the garage and the money I had to pay earlier that stressed me. The breathing one [Breath watch] I find easy to do on the spot, breathing and watching myself breathe calmed me down then I could call back the garage and check I had got the right price in my head…* (SH4) *The breathing technique is particularly good especially before going to sleep at a night, helps ‘get off quicker’ …* (LI3, Male, 62)
Intention to keep using the techniques in future	*Yes, yes I think so yes. Maybe not as often as I am now but I will keep doing it yes because I’m doing it every day at the moment. But I definitely shall.* (CA6) *I probably wouldn’t do it as often… it’s all about organizing your life and now like, obviously I am starting my own business I’ll also allow myself time to do other things like relax… because it really does help to do relaxation, I think it’s one of those supportive things that brings everything to a level*. (ST3) *I should be doing relaxation. Yeah, I will carry on using them [techniques].* (ST7, Female, 55)

Among survivors who participated in the T2 discussion/interviews, a general pattern was found that the techniques were user-friendly and easy to practice. This was substantiated with the fact that no participant sought additional help or raised concerns during the telephone contact between T1 and T2. One participant even practiced these techniques on the train. One participant, however, preferred listening to music to using the techniques- ‘if someone’s talking to me I can’t relax.’ (SH2). Each technique requires approximately 3 min including practice time and the DVD format made practicing much easier. Participants also positively commented on the fact that these techniques have multiple components. It offers alternatives for the individual if one technique was not feasible to do. Participant BR4 (with communication difficulties) stated that the techniques ‘picked her’ and she perceived them to be the ‘easiest to do’ together. Although most participants in the group discussions/interviews reported that the techniques were acceptable and easy to use, making the time to practice ‘twice a day, five times a week’ was relatively difficult for some. Some participants suggested that if they had been instructed to practice less often, it would have been more feasible to build the practice into their busy lives.

Another general pattern reported by participants in the T2 discussion/interviews was they generally felt practicing the techniques helped calm them down. The techniques relaxed their body (e.g., fall to sleep quicker), and took their minds away from worrying situations. Some reported they were more prepared to deal with specific stressful events such as interacting with others. One survivor who used the techniques once or twice a week, stated that she felt less anxious during practicing, yet ‘things start back up again’ due to a busy family life and the fear of another stroke. The theme on intending to keep using the techniques was consistent across the datasets of the focus groups and interviews. The more frequent participants used the techniques ‘the easier it gets to do’ (CA6), and ‘kept going’ with the techniques have helped (SH4). However, participants would prefer practicing less often so it would be easier to incorporate the practice into their lives. Other survivors believed they should incorporate the practice into their schedules as the techniques did help them relax.

## Discussion

### Study 1

We asked stroke survivors to choose their preferred techniques from a set of mindfulness and relaxation techniques delivered on a DVD. Thinking of a nice place, breath watch, positive emotions, and body relaxation were the most favored four as they were perceived easy to do and beneficial. Participants did not like the counting, word repetition or body movement techniques as they did not work. They were also difficult to concentrate on and hard to do particularly for survivors with communication difficulties. Our results are comparable to a previous study that used similar psychological techniques among a sample of patients with chronic obstructive pulmonary disease (COPD) (Hyland et al., 2016). Like stroke survivors, participants with COPD preferred thinking of a nice place and body relaxation. However, counting and word repetition were also favored, whereas the technique of breath watch was not. This suggests that the nature of the illness and disability determines what techniques patients find easy or difficult as well as the patient’s different needs and preferences. Interventions such as psychological techniques vary between diseases and should be tailored to the target patient group (Hardcastle and Hagger, 2016). It is important to make interventions meaningful and accessible for stroke survivors, especially those with communication difficulties.

### Study 2

We tailored the four favored techniques in line with suggestions from participants in Study 1 and re-filmed them into a new DVD. We tested feasibility and acceptability of these modified techniques with a new sample of stroke survivors including some with communication difficulties. The techniques were generally perceived as feasible and acceptable to use, and beneficial to participants. Nonetheless, a less frequent practice time would be more practical for survivors.

### Feasibility

Feasibility was considered in terms of practicality of delivery, retention and adherence rate. During the telephone contact, no concerns were raised and no participant sought additional help during the study. Participants found the DVD format made practice easier. There were no issues with following the techniques using the DVD at home. We acknowledge, however, the use of DVD as the only delivery mode could have potential challenges (e.g., no access to a DVD player). In fact, having access to a DVD player was requested for participants in a feasibility study on exercise DVD for people with traumatic hand injury (Kingston et al., 2014). This suggests the benefit of having a combined delivery mode, although a written brochure might be challenging for survivors with communication difficulties in this study. Future studies could implement these techniques via contemporary technologies such as online video as well as a DVD.

Our retention rate of 63.2% (24/38) at T2 was similar to a recent feasibility study which recruited stroke participants through healthcare professionals (Patel et al., 2018). Nevertheless our rate was lower than a randomized, controlled pilot study using self-help autogenic relaxation CD (Golding et al., 2015). It could be due to the fact that our T2 data collection and focus group discussions were scheduled within a support group meeting. We would not have their T2 data if participants did not attend the scheduled group meeting. Current evidence suggested that using strategies such as arranging frequent contacts and visits at regular intervals would enhance the retention rate (Abshire et al., 2017). Therefore, we might be able to increase our retention rate by contacting and/or visiting people who did not attend T2, but that was not feasible due to time and resource constraints.

The techniques were practiced less often than the twice-a-day, 5-days-a week instruction. Based on the 50% returned and completed diary data, those who filled in the diary practiced approximately once daily over a 4-week period. This was substantiated with comments from some participants in the focus groups/interviews, suggesting practicing ‘once a day’ would be more feasible. However, it has to be born in mind that only half of the participants at T2 returned the practice diary.

### Acceptability

The techniques were perceived user-friendly and acceptable for stroke survivors who participated in the focus group discussions/interviews. Participants also commented on the particular benefit of having a combination of different techniques: they could select the one(s) that suited them best. This provides support to the suggestion that techniques that have multiple component as opposed to being single-component are better suited to patients and more beneficial (Spence et al., 1999; Hyland et al., 2016).

Another element of acceptability is the amount of effort and time required to practice as instructed (i.e., burden) (Sekhon et al., 2017). Over time participants found it gets easier to practice, yet they felt practicing “twice-a-day, 5-days-a week” required much effort and time. A less burdensome and more practical frequency might be “once a day,” which was comparable to that adopted in a study using a single component autogenic relaxation technique (Golding et al., 2015). Although the delivery format was different in their study (i.e., CD), our techniques consist of a form of autogenic relaxation (body relaxation). Our participants were a similar age and had their stroke also a relatively long time ago. It is possible that our sample would have found it more acceptable to practice these techniques once daily for four to 6 weeks.

Even with less practice frequency, most participants in the focus groups/interviews still reported the techniques beneficial. Continued practice helped them feel less anxious and prepared some to cope better with stressful situations. The techniques distracted them from worrying thoughts and generated a relaxing psychophysiological state (Langer, 1989; Benson and Klipper, 2000). Although a larger study, preferably a controlled trial, is needed to ascertain benefit beyond using any DVD, comments from participants provided preliminary evidence of the perceived effectiveness of using these techniques- another element of acceptability (Sekhon et al., 2017). This provided further support to the benefit of tailoring techniques to stroke survivors including those with communication problems (Intercollegiate Stroke Working Party [ISWP], 2016).

It was promising to see that participants in the focus group discussions/interviews intended to continue using these techniques after the study, although a less frequent practice was preferred. Indeed, long term benefits of regularly using mindfulness and relaxation techniques have been suggested (Benson and Klipper, 2000; Golding et al., 2017). Once learnt, these techniques can be applied rapidly and in practically any situation (i.e., in this study, practice on the train or without the DVD) (Öst, 1987). However, when interpreting our findings, we need to be mindful that over a third of participants did not participate at T2 and were missing from the focus group discussion/interviews.

### Limitations

Although participants were recruited from different geographical locations, all of them self-reported as White British. Therefore, our sample is not representative of United Kingdom stroke survivors.

We did not use formal measures to identify cognitive impairments or the severity of communication difficulties of our participants. Thus, the participants were largely self-selected. Volunteers were recruited via stroke survivors’ groups. It is possible that this ‘self-selected’ group of study participants may differ to those stroke survivors who did not volunteer to participate. Our participants also had their stroke several years ago. It is likely that they have already made recovery which was suggested by their moderate to high level of daily living and psychosocial functioning. In many ways this is probably the characteristics of active members of many support groups. It is, therefore, unknown how the findings might apply to those who are from other ethnic groups or less motivated to take part in research, and those who have been recently discharged from hospitals.

The low retention rate of participants at T2 and low rate of returned diaries also needed to be acknowledged. Although the return rate depends on the survivors attending T2, we had only 50% of returned and completed diaries. Unfortunately, we did not know why the diaries were not returned. One explanation is that self-report diaries may not be most effective in tracking participants’ engagement with home practice. However, this needs to be explored further in future research which also might consider more objective methods to record adherence. Future studies should also implement further strategies to increase the retention rate of participants (Abshire et al., 2017).

## Conclusion

We have addressed a substantial clinical need by making psychological techniques accessible and meaningful for the target patient group. The self-help mindfulness and relaxation techniques in our studies were selected by stroke survivors (Study 1) and further adapted to suit their needs, including the needs of those with communication difficulties. It is encouraging to see that participants in Study 2 focus groups/interviews generally perceived the techniques feasible and acceptable to use as a self-help approach. Although practiced less often than instructed, participants still reported the techniques useful and beneficial. Nonetheless, the practice frequency needs to be reduced to make it less burdensome and more practical for participants. Our results provide a good foundation and a useful first step for future studies to investigate the effectiveness of tailored mindfulness and relaxation on reducing anxiety and stress among community stroke survivors. Further research should aim to recruit a more heterogeneous and representative sample as well as implementing further strategies to increase the retention rate.

## Author Contributions

XW wrote the manuscript, designed studies 1 and 2, and conducted study 1. CS collected and analyzed data for study 2 for her Master project. LA co-supervised CS’s project with XW and revised the draft manuscript. MH is the demonstrator on the DVD films used in the paper. He also revised the draft manuscript for its structure. All authors participated in the revision of the draft and in the final approval of the version to be published.

## Conflict of Interest Statement

The authors declare that the research was conducted in the absence of any commercial or financial relationships that could be construed as a potential conflict of interest.
